# Hydrophilic extract of* Pistacia vera* pericarp protects against phenylhydrazine–induced hepatotoxicity and hemolytic anemia

**DOI:** 10.22038/AJP.2024.23925

**Published:** 2024

**Authors:** Fatemeh Amin, Najmeh Parvaz, Nahid Askari, Morteza Khademalhosseini, Sakineh Khanamani Falahatipour, Iman Fatemi, Fatemeh Khajehasani, Zahra Taghipour, Soudeh Khanamani Falahati-pour

**Affiliations:** 1 *Physiology-Pharmacology Research Center, Research Institute of Basic Medical Sciences, Rafsanjan University of Medical Sciences, Rafsanjan, Iran*; 2 *Department of Physiology and Pharmacology, School of Medicine, Rafsanjan University of Medical Sciences, Rafsanjan, Iran*; 3 *Department of Clinical Biochemistry, Faculty of Medicine, Rafsanjan University of Medical Sciences, Rafsanjan, Iran*; 4 *Department of Biotechnology, Institute of Sciences and High Technology and Environmental Sciences, Graduate University of Advanced Technology, Kerman, Iran*; 5 *Department of Pathology, Medical School, Rafsanjan University of Medical Sciences, Rafsanjan, Iran*; 6 *Department of Pharmacology, Faculty of Veterinary Medicine, University of Tehran, Tehran, Iran*; 7 *Research Center of Tropical and Infectious Diseases, Kerman University of Medical Sciences, Kerman, Iran*; 8 *Department of Radiology, Afzalipour Medicine School, Kerman University of Medical Sciences, Kerman, Iran*; 9 *Department of Anatomy, Medical School, Rafsanjan University of Medical Sciences, Rafsanjan, Iran*; 10 *Pistachio Safety Research Center, Rafsanjan University of Medical Sciences, Rafsanjan, Iran*

**Keywords:** Anemia, Hepatoprotective, Pistachio, Liver, Antioxidant

## Abstract

**Objective:**

*Pistacia vera* is commonly used in traditional medicine to treat various disorders. This study aims to investigate the anti-anemia and hepatoprotective effects of *Pistacia vera* pericarp extract (PVPE) in a rat model of phenylhydrazine (PHZ)-induced anemia.

**Materials and Methods:**

PVPE was prepared using the maceration method. The extract was administered at doses of 20, 80, and 160 mg/kg for 28 days to normal and PHZ-treated rats. The effects of PVPE were evaluated in terms of changes in biochemical, histological, hematological, and molecular biomarkers in the liver and blood.

**Results:**

Administration of PVPE to the anemic animals significantly restored these deleterious effects on hematological parameters compared to the anemic group. Kupffer cell activation was seen in the liver tissue of the anemic rats. Administration of PVPE mitigated these deleterious effects.

**Conclusion:**

PVPE has potent antioxidant activity and may represent a promising treatment for anemia and liver protection in clinical settings.

## Introduction

Anemia is a blood disorder with various types and it is caused by different factors. Hemolytic anemia is the most common type caused by hemolysis, a pathophysiological process that reduces the half-life of erythrocytes or occurs within red blood cells (Barcellini and Fattizzo, 2015). Hemolytic anemia is caused by genetic and environmental factors (Erasmus and Olukoga, 1990), and various hemolytic agents can induce hemolysis in animal models (Frimat et al., 2019). Phenylhydrazine (PHZ) is a toxic chemical that causes hemolytic anemia and injuries to the kidney, liver, and spleen (Kale et al., 2019). PHZ is also used to induce hemolysis in models due to its strong oxidant properties (Lee et al., 2014). There is a strong correlation between oxidative stress and hemolytic anemia.

There are various treatment options for anemia, including home remedies and drugs. Herbal drugs may provide a new avenue for developing drugs to treat hepatic complications of anemia due to their antioxidant effects (Rizwan et al., 2019; Ashraf et al., 2018; Khalid et al., 2018). *Pistacia vera* is a medicinal plant that belongs to the Anacardiaceae family (Tomaino et al., 2010) and is widely grown in West and Central Asia, especially in Iran, which is a major gene center for this plant (Ehsani et al., 2017). Different parts of *P. vera* have therapeutic properties such as anti-inflammatory, antioxidant, immunomodulatory lipid-lowering hypoglycemic, and hepatoprotective effects (Agouni et al., 2009; Hakimizadeh et al., 2021). For example, anthocyanins, which are main components of pistachio pericarp, have shown anti-inflammatory, antioxidant, and anti-carcinogenic effects (Hou et al., 2005). Catechins, which are phenolic mixtures with antioxidant properties, are also present in pistachio pericarp and have been shown to reduce the oxidation of low-density lipoproteins (Basu and Lucas, 2007).

Considering the unique antioxidant properties and chemical composition of *P. vera*, the current study aimed to investigate the effect of *P. vera* pericarp aqueous extract (PVPE) on hemolytic anemia and liver damage in rats treated with PHZ.

## Materials and Methods

**Preparation of PVPE**

**Figure 1:**
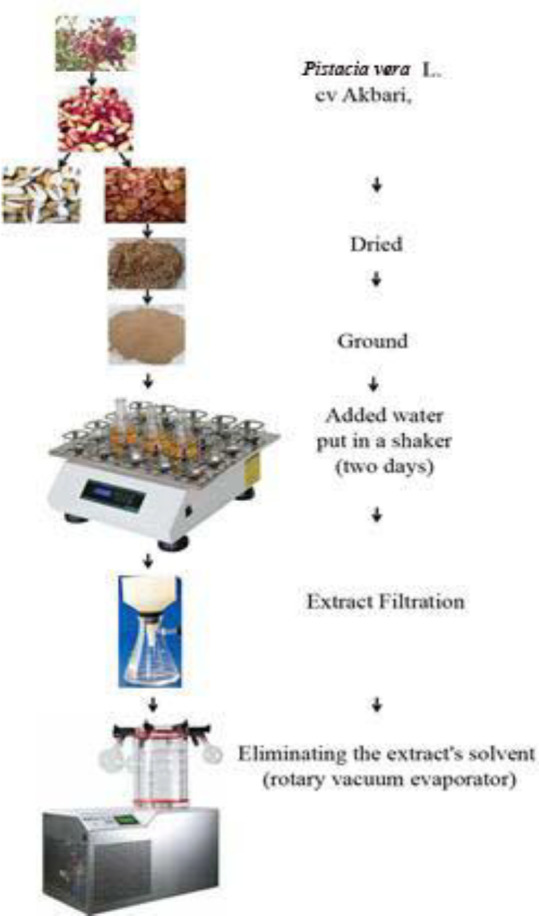
The extraction methodology

In this study, fresh *P. vera* L. cv Akbari that had not been exposed to any toxic sprays was used. The plant was identified by a horticultural specialist (Dr. Ali Tajabadipour) and a voucher sample (AK212) was obtained. The pistachio pericarps were dried in the shade, stored in a dark place before being ground into a powder and stored at -20°C until needed. To prepare the extract, 50 g of powdered pistachio pericarp was mixed with 100 ml of water and shaken for two days at 130 rev/min. The extract was filtered using Wattman No. 1 filter paper, and the solvent was removed using a rotary vacuum evaporator with a water bath temperature of 60°C. The resulting powder was stored at -20°C until needed (Figure 1). 

**Determination of PVPE polyphenol content and antioxidant activity**

The total phenolic content of PVPE was measured using the Soland and Laima method by reading the absorbance at 725 nm using a spectrophotometer (Soland and Laima, 1999). The total phenols content is presented as mg gallic acid/g. The total flavonoid content was measured using a colorimetric assay with an aluminum chloride reagent by reading the absorbance at 510 nm using a spectrophotometer. 

**Total flavonoid content**

The total flavonoids content is presented as mg/L (Zhishen et al., 1999). The Ferric Reducing Antioxidant Power (FRAP) assay was conducted by mixing 200 µl of PVPE with 1.8 ml of FRAP reagent and storing the mixture at room temperature for 30 min. The absorbance of the reaction mixture was measured at 593 nm using a spectrophotometer, following the method described by Benzie and Strain in 1996 (Benzie and Strain, 1996).

**Assay of 2, 2-diphenyl-1-picrylhydrazyl (DPPH)**

PVPE radical scavenging activity was assessed by measuring the inhibition of a certain volume (3 ml) of DPPH solution by 1 ml of the extract (Benzie and Strain, 1996). The absorbance of the reaction mixture was read at 517 nm using a spectrophotometer, following the method described by Mishra et al. (Mishra et al., 2012).

**Animal**

A total of 60 male Wistar rats weighing 200±5 g were obtained from the animal house of Rafsanjan University of Medical Sciences. The rats were housed under optimized conditions of temperature (22±3°C), ventilation, and light (12 hr light and 12 hr dark).

**Ethical guidelines**

The animal studies in this project were authorized by the Animal Ethics Committee of Rafsanjan University of Medical Sciences and carried out in accordance with the Guidelines for the Care of Laboratory Animals in Research (IR.RUMS.REC.1397.219).

**Induction of anemia**

PHZ was dissolved in sterile Phosphate Buffered Saline (PBS) at a concentration of 2 mg/ml, and anemia was induced by intraperitoneal injection of 60 mg/kg body weight of PHZ for two days. After 24 hr, rats with a hemoglobin concentration (HGB) <11.5 g/dl were considered anemic, based on the method described by Fernández et al. (Fernández et al., 2010).

**Experimental groups**

The rats were randomly divided into six groups of ten as follows: (1) Control group: rats in this group received 10 mg/kg of orally administered distilled water for 28 days; (2) PVPE 80 group: rats in this group received 80 mg/kg of orally administered PVPE for 28 days; (3) Anemic group: rats in this group were rendered anemic as described above; (4) Anemic+PVPE 20 group: anemic rats treated with 20 mg/kg of orally administered PVPE for 28 days; (5) Anemic+PVPE 80 group: anemic rats treated with 80 mg/kg of orally administered PVPE for 28 days; and (6) Anemic+PVPE 160 group: anemic rats treated with 160 mg/kg of orally administered PVPE for 28 days.

**Sampling**

At the end of the study (day 29), body weights of the rats were determined, and they were sedated with an intraperitoneal injection of 5 mg/kg xylazine and 50 mg/kg ketamine. Blood samples were taken from the hearts of the rats, and plasma samples were collected in EDTA tubes for evaluation of osmotic resistance of red blood cells and hematological parameters. Serum samples were collected in plain tubes for biochemical analysis, following the method described by Onyeabo et al. (Onyeabo et al., 2017). The rats were then decapitated and their livers were separated, weighed, and washed with saline. A portion of all samples was treated in 10% phosphate-buffered formalin for histological examinations, while the other portion of the tissue was homogenized (1/10 w/v) in ice-cold Tris-HCl buffer (0.1 M, pH 7.4) for biochemical assessments.

The relative liver weight was calculated using the following formula: 

Relative liver weight = liver weight (g) / body weight (g).

**Osmotic resistance of red blood cells**

The osmotic resistance of red blood cells was evaluated using the method described by Redondo et al. (Redondo et al., 1995). Briefly, red blood cell lysis was induced using a hypotonic solution. Saline solutions ranging from 0-9% were prepared and 50 µl of blood samples was added to each solution. The mixtures were shaken and incubated for 60 min before being centrifuged at 3000 rpm for 5 min. The absorbance of the supernatants was determined at 540 nm using a spectrophotometer. The percentages of hemolysis were calculated as follows: Absorbance test/absorbance full hemolysis. Full hemolysis was induced using distilled water, and the hemolysis curve was generated using different concentrations of sodium chloride.

**Hematological parameters**

Hematological parameters, including red blood cell count (RBC), mean corpuscular volume (MCV), hemoglobin concentration (HGB), mean corpuscular hemoglobin (MCH), and platelet count (PLT), were evaluated using an automatic cell counter (KX-21, Sysmex Corporation, Kobe, Japan).

**Biochemical analysis**

The concentrations of direct bilirubin (Bili D), total bilirubin (Bili T), and iron (Fe), and the serum activities of alanine aminotransferase (ALT), aspartate aminotransferase (AST), and alkaline phosphatase (ALP) were determined using Pars Azmoon's commercial kits (Tehran, Iran) and an automated analyzer (Biotecnica instrument, Italy).

**Oxidative and antioxidant parameters:**

The activity of superoxide dismutase (SOD) was evaluated by mixing 0.5 g/ml of protein from the hepatocyte suspension with sodium pyrophosphate buffer, phenazine methosulphate, and nitro blue tetrazolium chloride. The reaction was initiated by adding nicotinamide adenine dinucleotide reduced disodium salt (NADH), and the reaction mixture was incubated at 30°C for 90 sec before being stopped with 1 ml of glacial acetic acid. The absorbance was measured at 560 nm, following the method described by Karimi et al. in 2020 (Karimi et al., 2020). Catalase (CAT) activity was evaluated by mixing 5 g of protein from the hepatocyte suspension with 2.1 ml of 7.5 mM H_2_O_2_, and the decrease in absorbance at 240 nm was monitored spectrophotometrically for about 10 min at 25°C (Haybar et al., 2019). The level of malondialdehyde (MDA) was determined using the method described by Buege and Aust for hepatic lipid peroxidation (Buege and Aust, 1978). The spectrophotometric thiobarbituric acid reactive substances (TBARS) test is a method that measures malondialdehyde, which is a marker of lipid peroxidation. The tissue samples were heated for 15 min in a mixture of trichloroacetic acid and hydrochloric acid with thiobarbituric acid (TBA), and the absorbance was measured at 535 nm (Dehnamaki et al., 2019).

**Histopathological examination**

The fixed liver samples were processed in a tissue processor, embedded in paraffin wax, and cut into 5 µm thick sections using a microtome. The sections were stained with hematoxylin and eosin to detect pathological lesions and with Prussian blue staining to detect Kupffer cell activation. The slides were examined under a light microscope by a blind pathologist (Muller, Germany, model BioNet).

**Statistical analysis**

The data were organized in Excel and analyzed using one-way ANOVA and Tukey's HSD *post-hoc* test in SPSS (V. 21) (Windows-SPSS, Inc. Chicago, IL, USA). The results are presented as means±SEM and a p<0.05 was considered significant. 

## Results

**PVPE polyphenol content and antioxidant activity**

The total phenol content was 21.77 mg gallic acid/g and the total flavonoid content was 0.0096 mg/L. The average value of the antioxidant activity determined by FRAP and DPPH for PVPE was about 100.85 (mM ferrous equivalent) and 206.25 (µg/ml), respectively (Table 1). These data demonstrate that PVPE has a large amount of phenol and flavonoid mixes, as well as considerable antioxidant activity.

**Table 1:** Polyphenol content and antioxidant activity of PVPE

Total phenolic content (mg gallic acid/g)	Total flavonoid content (mg/L)	FRAP assay (mM ferrous equivalent)	DPPH assay (µg/ml)
21.77	0.0096	100.85	206.25

Data are expressed as Mean±SEM (n=10). Values with ^a ^and ^b^ indicate significant difference (p<0.05) in comparison to the control and anemic rats respectively. Statistical analysis was carried out using one-way ANOVA. PVPE:Pistacia vera pericarp extract

**Table 2:** Effect of PVPE at different doses on relative liver weight

Group	Control	PVPE 80	Anemic	Anemic+PVPE 20	Anemic+PVPE 80	Anemic+PVPE 160
Relative liver weight (g)	3.32±0.58	3.01±1.02	2.56±2.05^a^	2.97±0.21^b^	3.06±0.02^b^	3.10±0.36^b^

Data are expressed as Mean±SEM (n=10). Values with ^a ^and ^b^ indicate significant difference (p<0.05) in comparison to the control and anemic rats respectively. Statistical analysis was carried out using one-way ANOVA. PVPE:Pistacia vera pericarp extract

**Relative liver weight**

A significant difference was observed in the relative liver weight between the anemic group and the controls (Table 2). Administration of PVPE to the anemic animals (at all three doses) increased the relative weight significantly compared to the anemic group in a dose-response manner. Moreover, treatment of control animals with PVPE at the 80 mg/kg dose had no effect on this index.

**Osmotic resistance of red blood cells**

**Figure 2:**
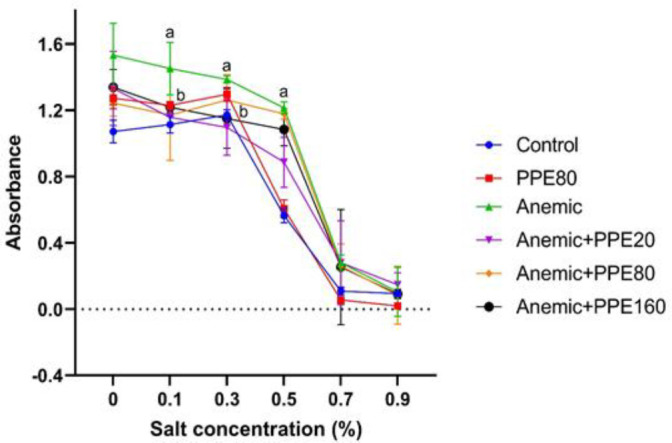
Effect of PVPE at different doses on the osmotic resistance of RBC in PHZ-induced anemic rats. Data are expressed as Mean±SEM (n=10). Values with a and b indicate significant difference (p<0.05) in comparison to the control and anemic rats respectively. Statistical analysis was carried out using one-way ANOVA.

There was a significant increase in the absorbance of the osmotic resistance test in the anemic group compared to the controls, indicating lower RBC osmotic resistance in the anemic group (Figure 2). PVPE administration at the 160 mg/kg dose significantly reduced the absorbance of the osmotic resistance test compared to the anemic group, indicating higher RBC osmotic resistance after treatment of anemic animals with 160 mg/kg PVPE. Moreover, treatment of control animals with PVPE at the 80 mg/kg dose had no effect on this index. 

**Hematological parameters**

Table 3 shows the effect of PVPE on different hematological parameters. Induction of anemia with PHZ reduced the values of RBC, HGB, HCT, and PLT, but increased the values of MCV and MCH in the anemic group compared to the controls.

Administration of PVPE to the anemic animals (at all three doses) significantly restored these deleterious effects on hematological parameters compared to the anemic group in a dose-response manner. Moreover, treatment of control animals with PVPE at the 80 mg/kg dose had no effect on these indices.

**Biochemical analysis**

PHZ induced significant changes in the serum levels of Bili D, ALT, AST, ALP, and Fe compared to the controls (Table 4). PVPE administration at the 20 mg/kg dose reduced the levels of Bili T and ALT, but increased the levels of Fe in the anemic animals, while at the dose of 80, it reduced the levels of Bili T, Bili D, ALP, and ALT, but increased the levels of Fe in anemic animals. In addition, administering 160 mg/kg PVPE to anemic animals significantly improved their condition.

**Table 3:** Effect of PVPE at different doses on hematological indices

Group Parameter	Control	PVPE 80	Anemic	Anemic+PVPE 20	Anemic+PVPE 80	Anemic+PVPE 160
RBC (*10^6^µl)	8.72±0.4	8.31±0.32	3.98±0.37^a^	7.91±0.31^b^	7.44±0.5^b^	7.99±0.74^b^
HGB (g/dl)	16.07±0.91	15.75±1.25	11.58±0.71^a^	15.44±1.14^b^	14.83±1.31^b^	15.06±0.89^b^
HCT (%)	41.67±0.73	41.03±1.79	36.2±2.54^a^	39.96±1.58^b^	38.17±2.8^a, b^	39.12±2.49^b^
MCV (fl)	47.83±1.46	49.4±1.44	91.35±7.14^a^	50.22±1.79^b^	51.27±0.59^b^	49.52±1.79^b^
MCH (pg)	18.43±0.21	18.95±1.07	29.18±1.72^a^	19.4±1.36^b^	19.9±0.40^b^	19.12±1.52^b^
PLT (*10^3^µl)	931.33±82.25	817.25±164.72	639.5±92.11^a^	765.6±119.53^b^	883.33±244.23^b^	836.8±433.54^b^

Data are expressed as Mean±SEM (n=10). Values with a and b indicate significant difference (p<0.05) in comparison to the control and anemic rats respectively. Statistical analysis was carried out using one-way ANOVA.

**Table 4:** Effect of PVPE at different doses on serum biochemical parameters

Group Parameter	Control	PVPE 80	Anemic	Anemic+PVPE 20	Anemic+PVPE 80	Anemic+PVPE 160
Bili T (mg/dl)	0.44±0.00	0.44±0.00	0.45±0.04	0.39±0.02^a, b^	0.39±0.01^a, b^	0.40±0.01^a, b^
Bili D (mg/dl)	0.08±0.01	0.09±0.01	0.12±0.02^a^	0.09±0.00	0.08±0.01^b^	0.08±0.15^b^
AST (U/L)	123.00±8.54	139.00±11.39	181.70±8.28^a^	168.30±6.75^a^	157.30±13.24	139.00±5.07^b^
ALT (U/L)	83.67±5.36	79.67±7.84^a^	153.00±37.00^a^	80.33±13.17^b^	80.67±6.37^b^	77.00±12.29 ^a, b^
ALP (U/L)	635.70±34.51	538.70±23.11	857.70±32.50^a^	680.70±83.65	628.00±54.50^ b^	646.70±23.42^b^
Fe (µg/dl)	123.33±7.13	167.00±10.44^a^	114.50±5.50^a^	197.00±67.43^a,b^	161.33±14.19^a, b^	150.00±35.01^a, b^

Data are expressed as Mean±SEM (n=10). Values with a and b indicate significant difference (p<0.05) in comparison to the control and anemic rats respectively. Statistical analysis was carried out using one-way ANOVA.

**Antioxidant parameter**

**Figure 3:**
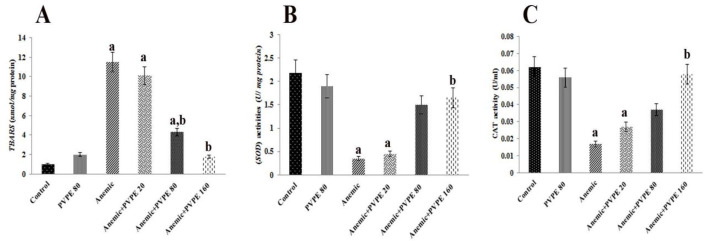
Effect of PVPE at different doses on antioxidant parameter. Data are expressed as Mean±SEM (n=10). Values with ^a^ and ^b^ indicate significant difference (p< .05) in comparison to the control and anemic rats respectively. Statistical analysis was carried out using one-way ANOVA.

The induction of anemia with PHZ significantly increased the levels of TBARS and the activities of SOD and CAT in hepatic tissues compared to the normal animals (Figure 3). Administration of PVPE at the 80 mg/kg dose only reduced the TBARS level, while at the dose of 160 mg/kg, it restored all of the deleterious effects of PHZ in the hepatic tissue. Moreover, treatment of control animals with PVPE at the 80 mg/kg dose did not affect these indices.

**Histopathological examination**

**Figure 4:**
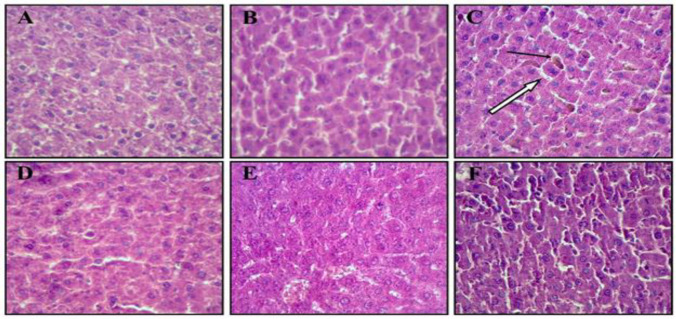
Histopathological studies of liver in rats after PHZ and PVPE administration (hematoxylin and eosin staining; 400X). (A) Control group, (B) PVPE 80, (C) Anemic group, (D) Anemic+PVPE 20 group, (E) Anemic+PVPE 80 group and (F) Anemic+PVPE 160 group. Black arrow: Kupffer cells; White arrow: Sinusoidal space.

**Figure 5:**
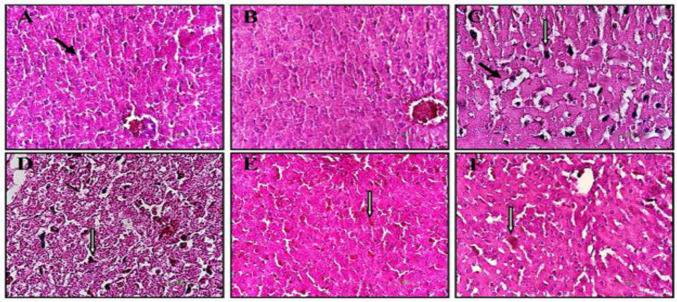
Histopathological changes scores of the liver in all experimental groups. Kupffer cells activation studies of liver in rats after PHZ and PVPE administration (Prussian Blue staining; 400X). (A) Control group, (B) PVPE 80, (C) Anemic group, (D) Anemic+PVPE 20 group, (E) Anemic+PVPE 80 group and (F) Anemic+PVPE 160 group. Black arrow: Sinusoidal space; White arrow: Activated Kupffer cells.

No significant pathological changes were observed in the control and treated groups (Figure 4A, B, D, E, and F). However, in anemic rats, the sinusoidal spaces were fairly wide, and the hepatocytes had a disordered arrangement (Figure 4C). There was no evidence of hepatocyte degeneration, inflammation, necrosis, or steatosis in the study groups. The Kupffer cells in the liver tissue of anemic rats were more activated (in terms of number and color) compared to the treated groups (Figure 5C, D, E, and F). 

## Discussion

The present research aimed to determine the protective impact of PVPE against PHZ-induced hemolytic anemia and hepatotoxicity in rats. We found that PVPE administration at the dose of 180 mg/kg for 28 days mitigated the deleterious effects of PHZ on the liver and blood parameters. We also demonstrated that PVPE has potent antioxidant properties potentially because of its phenolic and flavonoid compounds.

Assessing the body and organ weights is essential for toxicity studies and serves as a good and sensitive indicator for evaluating the overall health status (Tahraoui et al., 2010). In agreement with previous studies, we also demonstrated that PHZ reduced the relative liver weight, and PVPE administration increased this index in a dose-response manner. This effect can be associated with the presence of polyphenol and flavonoid-type antioxidants in the plant extract, which destroy free radicals.

Hemolytic anemia causes a wide range of cellular problems, including protein oxidation and membrane lipid peroxidation (Sung et al., 2013). PHZ administration leads to oxidative impairment of red blood cells due to reactive oxygen species (ROS) formation (Akara et al., 2021; Goorani et al., 2019; Lee et al., 2014). Our results demonstrated that PHZ reduces the RBC osmotic resistance in the anemic group, while PVPE administration at the dose of 160 mg/kg increased the RBC osmotic resistance. Phytochemicals of the *P. vera* protect red blood cells as antioxidants, which repair damaged cells (Turaskar et al., 2013). Numerous investigations have illustrated *P. vera* protection against oxidative stress-associated situations. It has been demonstrated that *P. vera* increases enzymatic activities against ROS and free radicals based on its antioxidant compounds (Hakimizadeh et al., 2019). However, to the best of the authors' knowledge, no reports are available about the impact of the pericarp extract of *P. vera* on oxidative stress problems (Hakimizadeh et al., 2021). Therefore, the ability of PVPE to adapt to the changes caused by PHZ in the current research can be related to the presence of factors such as phenols and flavonoids.

As reported previously, PHZ administration declines the concentration of Fe, HGB, HCT, PLT, and RBC, but increases the level of MCV and MCH in rats (Criswell et al., 2002; Onyeabo et al., 2017). The excess amount of Fe within the hepatocytes is observed after the PHZ-intoxication of rat models (Kim et al., 2009). Fe plays a crucial role in RBC formation and is a necessary ingredient of HGB. Fe has a remarkable effect on hematopoiesis. The availability of Fe from plant sources is lower than animal sources. The high levels of MCV and MCH observed in this study may be due to the deleterious effects of PHZ, which leads to macrocytosis caused by megaloblastic anemia (Akara et al., 2021). The lysis of the RBC caused anemia, which was naturally converted one week later by the regeneration of these blood cells because of the excess of reticulocytes. Today, people consume plant extracts to treat various diseases due to their fewer side effects and higher in comparison to synthetic drugs (Aktar and Foyzun, 2017). The present research illustrates that PVPE significantly increases the concentration of osmotic resistance of RBCs and the number of reticulocytes, as well as hemoglobin, one week after PHZ administration. Criswell et al. (2002) reported that reticulocyte counts elevated in PHZ-treated animals (Criswell et al., 2002). PVPE can enhance the number of reticulocytes by motivating the erythropoiesis process. Moreover, the present research indicates that the efficacy of PVPE might not be determined based on accessible Fe content alone, and it is possible that some other parameters have a role in Fe absorption in the body. Recent studies indicate that some herbs, plant parts, or their extracts can be used to treat disorders such as Fe deficiency anemia (Saha et al., 2018). For instance, *Trigonella foenum-graecum* leaves are sources of Fe and act as an effective supplement for anemia. Kone et al. reported that *Moringa oleifera* has beneficial effects in treating Fe deficiency anemia (Kone et al., 2012). More studies need to be conducted in the future to design a supplement made of *P. vera* pericarp that is not only rich in Fe content but also includes flavonoids, vitamin C, folic acid, tannins, and other components. 

Therefore, the body can store these substances to use them against anemia. Our aim is to develop a comprehensive supplement for the disease using extracts from the pericarp of *P. Vera* along with some essential elements. 

The liver is a vital organ that has many pivotal functions in modifying homeostasis, body maintenance, and detoxification (Hakimizadeh et al., 2022). In addition to PHZ's hemolytic activity, it is toxic to the liver. It is well established that serum liver enzymes such as ALT, AST, ALP, Bili T, and Bili D increase in hepatocellular abnormalities (Akara et al., 2021). Bilirubin is produced by the breakdown of RBC, which induces the release of hemoglobin in the spleen and liver, where Fe is released from hemoglobin together with biliverdin, which then gets transformed by biliverdin reductase to bilirubin and stays in the body. The liver eliminates bilirubin as a waste product from the blood (Fevery, 2008). After treating the animals using PHZ, the elevated serum ALT, ALP, AST, and Bili D levels indicated liver damage and hepatocellular problems (Park et al., 2019; Akara et al., 2021; Pourgholam et al., 2006). PHZ increased the production of bilirubin by causing inflammation in the liver and the bile duct, as well as hemolytic anemia. (Janghel et al., 2019). In line with previous studies, our results indicated that exposure to PHZ increased a rise in the ALP, AST, ALT, and Bili D concentrations compared to the control animals. In the present study, treatment of anemia in animals using PVPE demonstrated positive effects through biochemical and cellular pathways, including decreasing the serum markers such as ALT, AST, ALP, Bili T, and Bili D. It is well documented that herbs with antioxidant effects mitigate the PHZ-induced hepatotoxicity via modulating liver enzymes (Henneh et al., 2020). For instance, *Ocimum gratissimum*leaf extract has a protective effect against the PHZ-induced deleterious effects on the liver (Akara et al., 2021). On the other hand, Mehenni et al. reported that in hepatic injury,* P. vera *extract treatment reduces the levels of serum liver enzymes that are elevated by liver damage (Mehenni et al., 2016). They demonstrated that the leaf crude extract was more efficient in comparison to its fruit counterpart, which can be related to the higher amounts of phenolic components. Also, Mahdizadeh et al. recommended that *P. vera* reduces the raised liver enzyme levels in liver congestion and hepatotoxicity or toxic hepatitis (Mahdizadeh et al., 2015).

Previous studies have reported that PHZ damages hemoglobin and red blood cells by producing ROS and free radicals and triggering a cascade of inflammatory responses (Lee et al., 2014). Kupffer cell activation was involved in some intense inflammatory responses in the initial phase of the acute liver failure pathogenesis (Yang et al., 2014). In the current research, some changes in the liver tissue of the anemic rats were observed, including Kupffer cell activation, which was not observed in other groups treated with PVPE, indicating the antioxidant properties of PVPE. On the other hand, Goorani et al. also reported degeneration changes in the liver of PHZ-treated rats (Goorani et al., 2019), but in our study , no fatty changes were identified.

In conclusion, the higher nutritive value and functional properties of *P. vera* suggest its use in nutraceutics, medicines, and pharmaceutics. Our results confirm the therapeutic effect of *P. vera* in treating hepatic complications of anemia in traditional medicine. However, more research is needed to investigate the chemical constituents of this multipurpose plant and its pharmaceutical potential.
